# On the History of Quinine; the Preparations of That Article, and Their Use in the Treatment of Diseases

**Published:** 1851-02

**Authors:** Theodore S. Bell

**Affiliations:** Louisville, Ky.


### EDITORIAL DEPARTMENT.

*On the History of Quinine; the Preparations of that Article, and their Use in the Treatment of Diseases.* By Theodore S. Bell, M. D., of Louisville, Ky.

Nearly ten years ago, we communicated for the American Journal of Medical Sciences a report on the treatment of Intermittent fever, in which the following positions were advanced: 1. That Quinia may be administered in single doses of from 15 to 30 grs., at any period during the apyrexia, without any unpleasant consequences, and with the effect of arresting promptly the paroxysms; and that in some instances in which doses of a single grain of this remedy repeated at short intervals are with difficulty retained, five grains, or more, give rise to little or no uneasiness. Facts derived from an analysis of a large number of cases were submitted in support of these conclusions. The employment of Quinia, in the doses just mentioned, was not claimed as original. Other observers had reported results leading to similar conclusions. We were not, however, aware of this while the cases which we reported were in progress. Since the publication of the article referred to, Quinia has been administered much more freely than we then deemed safe, or should now venture upon, save in some desperate cases. Owing to the tendency to extremes, this invaluable drug, of late years, has been employed in doses as excessively large, as they were formerly inadequately small. In discovering that most of the dangers which once restricted its employment to particular periods, as well as in minute doses, are imaginary, it seems to be overlooked, by some, that any evils can possibly arise from it—that, like other remedies, it is capable of exerting a potency for evil, as well as good.

2. A second position advanced was, that, in the management of Intermittents, the specific remedy, the Quinia, should be promptly resorted to, without waiting for what is known as the "preparatory treatment." We contended that no preparation was needed, and that not only was time lost by the delay required for the operation of cathartics, emetics, and mercurials, but the cure was rendered thereby more difficult, and less complete. The plan of preparing the system in the reception of the Quinia is still enjoined by most, if not all systematic writers on practical medicine, and pursued, if we mistake not, by the majority of practitioners.

3. A third position was, that the sooner the paroxysms are arrested, the less is the liability to relapses. An opposite opinion has been advocated, and is still a popular belief, leading persons suffering from Intermittent fever, to permit the disease to go on unchecked, with the expectation that it will, in the end, "wear itself out."

4. A fourth position was, that by promptly resorting to the use of Quinia in the early stage of remitting fever, the progress of the fever, in many instances, is arrested, and the disease converted into an Intermittent which is readily curable.*

Ten years' experience has served to confirm, more and more, the correctness of the foregoing opinions.

Our attention has been called to the subject by the perusal of a highly interesting, and able memoir in the Louisville Medical Journal, the caption of which is prefixed to this article. Dr. T. S. Bell, one of the accomplished editors of the Louisville Journal, author of the memoir, in addition to various historical, and other facts relating to Quinia, enunciates, as the results of his own abundant experience, views similar to those set forth in the four positions which have just been stated. By premising these positions we do not mean to imply any connection between the article in which they were contained, and the opinions of Dr. Bell. Dr. Bell's views, indeed, as he states, were formed prior to the publication of that article, nor was the latter seen by him until his memoir was written. He mentions the latter fact, and takes occasion to refer to our communication in flattering terms of commendation.

A correspondence in views which are at variance with commonly received doctrines is always a source of gratification; and, in this instance, we assure Dr. B. that his own sentiments are reciprocated, and for the same reasons assigned by him, reasons which are much more pertinent in their application to himself. Believing that the practical views at which we have arrived, each by his own observations and reflections, independently of the other, will, ere long, be generally adopted by the medical profession, we confess that we have some personal interest in referring to the publication made by us on this subject, in the Am. Jour. of Med. Sciences, Oct. 1841, and this belief is, in part, our apology for so doing by way of introduction to some extracts from the memoir by Dr. Bell.

Passing by several pages of historical details which, however will well

* A fifth position might be added, viz., that if complications exist, the paroxysms should first be arrested, and the complications afterward attended to. The reverse has been, if it be not still the common practice.

repay perusal, we quote the following, simply adding that we trust the writer's caustic satire upon the *Hepatic pathology* will do something toward rendering it obselete—a consummation devoutly to be wished:

The writer of these remarks was taught in the lecture room, that the use of quinine was altogether unnecessary in the management of intermittent and remittent fevers. The doctrine seemed so clearly demonstrated, that it was as implicitly relied upon as if it had been written by Moses on Mount Sinai. No one, after a long experience with quinine or Fowler's solution, ever gave those remedies with more entire confidence in a favorable result, than I gave pills of calomel, aloes, and rhubarb with a certainty of purging off the periodical return of the cold stage. It was a matter of amazement that agues could be so remarkably stubborn, as I found them, but then I had been taught that they were often very stubborn and intractable under any treatment. And even when necessity forced a surrender of the simple and beautiful theory to the stubborn requirements of experience and observation, the theory was comforted, and the patient was victimized, with an error scarcely less pernicious than that which was abandoned—that error was, that there was an absolute necessity of preparing the system for the use of quinine. I had been taught that there was a viscus in the human economy, called the liver, which played such an active, omnipresent part in all diseases, that there seemed to be no room for any thing else in the human body. Its boundaries were very imperfectly understood. In a very large class of diseases of the brain, the liver was there; in the most of thoracic diseases, the liver was in the thorax; in nearly all abdominal diseases, the ramifications of the liver were to be distinctly traced. The brain, the lungs, the heart, pancreas, stomach, intestines, kidneys, uterus, bladder, etc., were mere satellites moving around a central sun, and that sun was the liver. In examining Dr. Stokes' daguerreotype of the Hepatic school of medicine, "in which the existence of almost every organ, except the liver, seems to be forgotten, and in which the creed seems to be that there is but one viscus, the liver, one source of disease, biliary derangement, and one cure, mercury;" it struck me, that I knew a man, whether in the body I shall not tell, or whether out of the body I shall not tell, who was covered by the picture as perfectly as any other sleeper ever was covered by a blanket. There seemed to be a necessity for looking up the other viscera, and for ascertaining whether they had any actual uses, and what were their conditions in health and disease. This was a work of labor, but the rewards have been very great, and the improvement has been so useful, that the exercises that produced it may be reasonably commended to others as conducive to the making up of the practitioner of medicine. The discovery of one additional viscus, where the existence of only one had been recognised before, is something in the way of progress; the full recognition of the movements and relations of all the viscera, in health and disease, is worth all the labor and perseverance that are necessary to its attainment.

It is somewnat singular with what tenacity an error frequently holds its ground in the practice of medicine. The necessity of putting the stomach and bowels in a special condition for the use of quinine, in the treatment of intermittent disease, and of remittent fever, is one that had such a feeble footstalk and is so palpable, that it is wonderful it could have lived long under the light of reason. The theory that gave the error much of its *animus*, was sufficient of itself to overthrow it. The first link in the chain of evils was said to be weakened action of the heart; this brought on congestion of the vena cava; this produced derangement of the liver, and that opened Pandora's box. And yet we were taught to permit this state of things to go on day after day, to remove congestion at night, and have it renewed the following day, or the day after, as the case might be, thus introducing into practical medicine the labors of Penelope! It is evident that each returning chill, each exacerbation of fever, must renew those derangements which physicians were endeavoring to remove, and that the proper mode of breaking up the concatenation of morbid phenomena would be found in that plan that promptly arrested the chill or the exacerbation of fever. And when the inquiry was made, what is there in the condition of the stomach and bowels, at the outset of periodical fever, that forbids the prompt use of quinine for its imediate arrest, it was impossible to give any satisfactory reason for delay. But custom was too strong, and the prejudices of early education are almost almighty. I broke through the meshes of early teaching with great difficulty, but got out with rapidity after the first attempt was made. A case of malignant chill occurred in this city, many years since, which created some noise at the time. I heard of the case with surprise, and upon inquiry, I was told that there was nothing unusual in the two chills that preceded the fatal one; that there was nothing whatever that induced a suspicion that the third chill would assume a malignant form. This aroused in me a determination never to permit a patient of mine to have a third chill, and this determination was strengthened soon after by the distressing case of a professional friend in the county of Jefferson. I had seen him but a few days before his illness, in full and vigorous health, apparently, and was surprised by a summons to visit him one evening, at his place in the country. I found him in the tenth hour of a malignant chill, presenting all the phenomena of a collapsed case of cholera. There was not a symptom of that stage of cholera absent from this case of malignant chill. Upon inquiry, I found that the victim in this case had paid little or no attention to the first and second chills, but had attended to his professional duties during the intermissions, and had trusted to an emetic, on the morning of the third chill, for arresting the ague fit. The emetic failed, and reaction never came on.

The two cases of malignant chill thus brought before my observation effected a thorough change in my views of the correct treatment of intermittent fever, and I have had no reason to regret the change. The rule adopted then, and persevered in ever since, was that the presence of intermittent fever was the exact preparation that required the use of quinine, and that the earliest administration that could be made of it, was the very best thing that could be done for the patient. Auxiliaries have sometimes been resorted to in this practice, but they were always subservient to the quinine—that is the chief and reliable remedy. In commencing this practice, which I was then persuaded was new, my intention was to amend the condition of the chylopoetic system after the intermittent malady was broken up, but in a long series of years, and in a great multitude of cases of all the various forms of intermittent and remittent fever known to this locality, I have rarely found it necessary to do anything after the ague was destroyed. The questions to be decided are: is it safe to administer quinine in intermittent and remittent fever, without preceding its use with emetics or purgatives? Is the patient likely to do as well under the immediate administration of quinine, as under the use of it, after emetics or purgatives? Experience and widely extended observation answer in the affirmative, and go yet farther, for they testify that under the immediate exhibition of quinine patients are not only in as safe a condition as under the preparatory treatment, but that they are infinitely safer. They escape prolonged sufferings, avoid dangerous lesions, and are free from the risk of the malignant chill. These are abundant reasons for preferring the prompt use of quinine in periodical disease, but the advantages are much more numerous than those I have named.

The following extract relates to the same subject:

It is unnecessary to dwell at length upon the fatal lesions that may result from delay in bieaking up the proximate cause of dangerous congestions. They are too familiar to all who have practiced in a malarious region. These dangers may not always result in perilous lesions, but even the morbid effects stand prominent enough, as beacons, to admonish a prompt removal of the periodical attack. Mere functional derangement, as a consequence of persistent intermittent fever, in many cases makes itself felt in all of the subsequent life of the patient. Truth, experience, observation, fidelity to the profession of medicine, and a faithful guardianship of the welfare of the sufferer, all imperatively require of the physician the utmost promptitude in arresting the chills. When I can cut the first paroxysms off in the middle of its efforts, I always do it, and never permit a second one to show itself, when I am called in time to interfere. As long as we are ignorant whether the attack of chills may terminate in the malignant variety; of what fatal lesions may occur; of what functional disorders may be left as heir-looms, by child's play with the disease, that length of time will physicians be called upon to use promptly the resources of their art in breaking up that state of things that is so frequently perilous, and the existence of which is always unnecessary. I have seen patients purged day after day for weeks, the physician apparently waiting on providence in the hope that something would turn up to justify the use of quinine. And when the state of the patient at length compelled the use of that medicine, the patient's system was not as well "prepared" for the use of the quinine, as it was before the scientific preparatory treatment was commenced, yet to their amazement the patient recovered without any evil effects from the quinine. I should think that such facts would have an effect upon the after-practice of the physician, but there are men who will walk through mud-holes in a well-beaten path, especially if the path has been beaten by themselves, rather than turn out of the road. Upon such the lights of experience shed their rays in vain; with them all the phenomena of disease, and recovery, are as accidental as the throws of the dice, and they forget that in practical medicine, fortunate numbers may turn up, if the dice are properly loaded. The man who does not improve under the teachings of experience is a pitiable object, but the physician who does not advance in such a school is baleful.

With respect to the use of Quinia in doses that formerly would have been deemed hazardous, the writer holds the following language:

Within the past few years a great change has taken place in the mode of administering quinine, especially, in intermittent fever. The practice formerly was to commence in the apyrexia, and give two grains of quinine every two hours until the return of the period for the recurrence of the paroxysm. This plan was supposed to be the only safe one; a great fear was felt of the stimulating effects of the medicine, and it was quite common "to watch the effects," as the phrase goes. Although many physicians stepped out of this well worn rut, the experience of the physicians of the United States army, in Florida, first gave prominence to the great superiority of administering quinine in a large and decided dose, at once. The paper, read on this subject before the National Institute, first taught the value of the practice, and it has steadily increased in the estimation of observant physicians, from that time to the present. Where an interval of from fourteen to eighteen hours can be found, a single dose of twenty grains of sulphate of quinine, administered to an adult, at the commencement of the apyrexia, and from four to six grains to a child, are the best means of managing the disease. This plan is quite as certain in its result, in arresting the paroxysm, as the divided portions of quinine were; the disturbance of the patient, the breaking of his sleep, and the consequent injury to the nervous system, are all avoided by the decided dose. A patient can take twenty grains of quinine, and rest secure against any further return of the chill.

The writer gives his experience in the use of Quinia in Remittent fever in the following paragraph:

In simple remittent fever I have generally pursued the course described in the treatment of intermittent fever. The great object is to make as perfect a remission as possible, and this can usually be done by cold ablutions of the body and spine; cloths with hot water in them, to the head, and a judicious use of the lancet. These means, combined with the administration of quinine, are all that I usually find necessary in the treatment of simple remittent fever, and the success has not only been gratifying, but I have been able greatly to facilitate the cure of such cases, in comparison with the former treatment. It is seldom that the lancet, purgatives or nauseants have to be used. The quinine may be used in the early periods of remittent fever, in the same way that it is given in the intermittent form. Of course, after serious inroads are made upon the healthy functions of the viscera, other means than the quinine must be resorted to, but even then, that medicine is an important adjunct.

The principle involved in the following extract we are satisfied is as true, as it is practically important in the treatment of intermittents. We have long entertained and acted upon the opinion that, by continuing the remedy for a long period after the paroxysms are arrested, the liability to relapse is lessened, and also that Quinia possesses prophylactic virtue. We are persuaded that we have repeatedly warded off a relapse by a timely resort to the remedy. Dr. B. cites his own case as follows:—

In 1835 I had an attack of intermittent fever, and became the subject of frequent relapses. They became so annoying that I consulted a number of medical friends, whose age and experience, I hoped, might furnish me some means to avoid the troublesome returns of the disease. They assured me that they knew of no medical means that would answer the purpose. It seemed to me that I was condemned to a life of suffering and inaction, and the subject occupied my thoughts almost constantly. At length I struck out an idea that ought to have been palpable at first. It is, that the quinine that interrupts the action of the cause of the paroxysm, if continued long enough, will remove the cause altogether, and I acted upon the thought at once in my own case. From that time to the present I have had no relapse, and as fifteen years have elapsed, I think there is little danger of one now, and that the principle has fairly tried itself. In taking quinine for this object, it is necessary to use it but once a day, in doses varying from three to ten grains. Under this course of treatment I have seen but little difficulty with relapses. Some years after this use of quinine, I was gratified in finding that Dr. Graves, of Dublin, was advocating the principle, which had been successful in my own case.

We have confined our quotations from the memoir by Dr. Bell to portions relating to the points embraced in our introductory remarks. The writer treats of the applicability of the Quinia to other diseases than Intermitting and Remitting fevers, viz., to Congestive fever, Yellow fever, Rheumatism, Neuralgia, Cholera, etc. His remarks upon these relations of the subject are valuable; but to do justice to the memoir we should transfer it entire to our columns, which our limits forbid.

